# Effect of humidification intervention combined with cluster nursing on ventilator-associated pneumonia in ICU patients

**DOI:** 10.3389/fpubh.2025.1669521

**Published:** 2026-03-20

**Authors:** Ying Liu, Juan Liu, Danfeng Zhang

**Affiliations:** 1 Nanjing Brain Hospital Affiliated to Nanjing Medical University, Nanjing, Jiangsu, China; 2 Sir Run Run Hospital, Nanjing Medical University, Nanjing, Jiangsu, China

**Keywords:** ventilator-associated pneumonia, ICU nursing care, humidification, cluster intervention, heat-and-moisture exchanger, mechanical ventilation

## Abstract

**Background:**

Ventilator-associated pneumonia (VAP) is a serious complication in mechanically ventilated patients, increasing morbidity, mortality, and antibiotic use. Although cluster nursing and humidification methods independently reduce VAP risk, their combined effect has not been rigorously evaluated.

**Objective:**

To determine whether combining dual humidification (heated humidifier plus heat-and-moisture exchanger) with a structured cluster nursing bundle reduces VAP incidence in Intensive Care Unit (ICU) patients. Methods: In this single-center cross-sectional observational study, 240 adult ICU patients expected to require ventilation for >48 h were observed under either enhanced care (dual humidification plus cluster nursing) or standard care. The cluster nursing bundle included head-of-bed elevation (30–45°), chlorhexidine oral care every 4 h, daily sedation breaks with weaning assessment, subglottic suctioning, enteral nutrition with prophylaxis, thrombosis prevention, and strict hand hygiene. The primary outcome was VAP incidence defined by CDC/NHSN criteria. Secondary outcomes included ventilator duration, ICU length of stay, antibiotic use, and mortality.

**Results:**

Ventilator-associated pneumonia incidence was significantly lower in the enhanced care cohort (10.0%) compared with standard care (30.0%) (*p* < 0.001), representing a 67% relative risk reduction (number-needed-to-treat = 5). Enhanced care patients had shorter ventilation duration (7.4 vs. 9.1 days, *p* = 0.003), shorter ICU stays (10 vs. 13 days, *p* = 0.01), fewer antibiotic days (6.8 vs. 10.7, *p* < 0.001), and delayed VAP onset (7.5 vs. 5.0 days, *p* = 0.04). Mortality was lower (18.3% vs. 25.8%) but not statistically significant. No tube occlusions or adverse respiratory events occurred.

**Conclusion:**

A dual humidification strategy combined with a high-compliance cluster nursing bundle significantly reduced VAP incidence and improved ICU outcomes. This cost-effective approach supports implementation to enhance patient safety and reduce antimicrobial resistance.

## 1 Introduction

Ventilator-associated pneumonia (VAP) is a serious infection acquired in the Intensive Care Unit (ICU), occurring in patients after ≥48 h of mechanical ventilation. VAP is classified into two types according to the onset of pneumonia: early-onset VAP, which develops within the first 4 days of mechanical ventilation, and late-onset VAP, which occurs ≥5 days after initiation of mechanical ventilation. It remains one of the most frequent nosocomial infections in critical care, affecting an estimated 20–36% of intubated ICU patients (1). Reported incidence rates vary from 5 to 40% of ventilated patients (2–16 episodes per 1,000 ventilator-days) depending on patient population, preventive practices, and diagnostic criteria (2). VAP imposes significant burdens: it prolongs mechanical ventilation and ICU stay, increases healthcare costs, and is associated with considerable morbidity and mortality. Attributable mortality from VAP has been estimated around 10% in general ICU populations, though crude mortality of VAP patients can range widely (24–76%) due to differing illness severities and pathogens (3). Notably, VAP induces substantial antibiotic utilization, accounting for roughly half of all antibiotic use in the ICU. This high antibiotic pressure contributes to the emergence of multidrug-resistant organisms, creating a vicious cycle of difficult-to-treat infections. Effective strategies to prevent VAP are therefore critical to improve patient outcomes and reduce antimicrobial resistance (4).

Multiple evidence-based interventions have been shown to reduce VAP risk. In particular, “cluster nursing” – the implementation of a care bundle of preventive measures – is recommended to prevent VAP (5). Numerous studies confirm that adhering to VAP bundles significantly lowers VAP incidence. For example, a 2023 meta-analysis of 36 studies (116,873 patients) found bundle implementation reduced VAP odds by ~58% (OR≈0.42) and also shortened duration of mechanical ventilation and ICU stay (6). Common bundle elements include elevating the head of bed (30–45°), daily sedation interruption and weaning assessments, diligent oral hygiene with antiseptics, prophylaxis for peptic ulcer and deep vein thrombosis, endotracheal tube cuff pressure management, and subglottic secretion drainage (7). A large systematic review reported that many studies achieving high compliance with such bundles saw VAP reductions over 50%, with some ICUs approaching zero VAP rates (8). Real-world quality improvement projects have mirrored these results: in one ICU, reinforcing bundle compliance and nursing education reduced VAP rates from 20.8 to 12.2%, while also decreasing ventilator days, tracheostomy rates, and hospital length of stay (9). Similarly, cluster intervention trials in China have demonstrated significant VAP reductions. Xiong et al. (9) reported that implementing cluster airway nursing in mechanically ventilated patients with respiratory failure lowered VAP incidence (research group 13.3% vs. control 26.9%, *p* < 0.01) (10). Li et al. (11) found an optimized cluster nursing strategy cut VAP incidence from 17 to 4% compared to standard care (12). These findings underscore that a multidisciplinary nursing bundle can dramatically improve VAP outcomes in the ICU.

Proper humidification of the ventilator circuit is another important consideration in VAP prevention. Intubation bypasses the upper airway, impairing natural heating and humidification of inhaled gasses. Inadequate humidification dries the airway mucosa and thickens secretions, compromising mucociliary clearance and increasing the risk of airway occlusion, atelectasis, and pulmonary infection. There are two primary humidification modalities in ICU practice: active heated humidifiers (HH) that warm and humidify inspired gas, and passive heat-and-moisture exchanger (HME) filters that conserve exhaled heat/humidity and filter microorganisms. It remains controversial which method is superior for VAP prevention (13). Some guidelines have recommended HMEs, noting that not using an HME is a risk factor for VAP. HMEs can reduce circuit condensate and act as a barrier to airborne pathogens, and recent clinical data associate HME use with lower VAP incidence (14). For instance, a large 2024 cohort study found that patients ventilated with HME filters had a significantly lower odds of VAP (OR ≈ 0.86) while those with active humidification had slightly higher odds (OR ≈ 1.14). The authors attributed HME benefits to less condensate and microbial filtration. Consistently, the lack of an HME was among independent risk factors for VAP in some analyses. However, other studies have found no significant difference in VAP rates between HH and HME when standard care is applied. A quasi-experimental trial in Barcelona (12) switched an ICU from HME to HH (with identical VAP-prevention protocols) and observed nearly identical VAP incidences (~5.7 vs. 5.8 per 1,000 vent-days) (12, 15). Thus, with high bundle compliance, humidification type alone might not drastically affect VAP. Each approach has trade-offs: HMEs raise dead space and can cause hypercapnia or tube occlusion if clogged with secretions, whereas HH systems require vigilant water management to prevent contamination and circuit breaks. There is interest in whether combining active and passive humidification could synergistically improve outcomes – for example, using a heated humidifier to ensure optimal moisture plus an HME filter at the airway for heat retention and microbial filtration. This combined humidification strategy, while not standard, might theoretically reduce VAP by mitigating both dryness and contamination. To date, evidence is limited on the efficacy of combined HH + HME use, and no RCTs have specifically examined it. We hypothesized that integrating a combined humidification approach with a nursing bundle would yield additive protection against VAP.

We conducted a cross-sectional observational study to evaluate the effect of combined humidification plus cluster nursing care on the incidence of VAP in mechanically ventilated ICU patients, compared to standard care. The primary outcome was the occurrence of ventilator-associated pneumonia (VAP), defined according to CDC/NHSN criteria as pneumonia developing >48 h after initiation of mechanical ventilation. Secondary outcomes included time to VAP, ventilator days, ICU length of stay, mortality, and antibiotic usage. We also monitored compliance with key care processes and any adverse events (like tube occlusions) related to the intervention.

## 2 Materials and methods

### 2.1 Design

This was a prospective cross-sectional observational study conducted in intensive care unit (ICU) of Nanjing Brain Hospital Affiliated to Nanjing Medical University. The study aimed to evaluate whether combining dual humidification (heated humidifier plus heat-and-moisture exchanger, HME) with a structured ventilator-associated pneumonia (VAP) prevention bundle could reduce VAP incidence and improve ICU outcomes compared with standard care.

### 2.2 Trial registration

The study was approved by the Ethics Committee of Nanjing Brain Hospital Affiliated to Nanjing Medical University (Approval No. 78872/NBH). The study complied with the Declaration of Helsinki and relevant national regulations.

### 2.3 Sample size

The intended sample size was 240 patients, providing 80% power to detect a 50% relative reduction in VAP incidence (from 30 to 15%) with a two-tailed *α* of 0.05, accounting for an anticipated dropout of less than 5%.

### 2.4 Participants

A total of 265 patients were screened, and 240 patients meeting the inclusion criteria were enrolled and allocated 1:1 to either the intervention or control cohort. Participants were stratified by admission diagnosis and illness severity (APACHE II scores) to ensure baseline comparability. All patients were managed by the same ICU nursing and physician teams throughout the study.

### 2.5 Eligibility criteria

Inclusion criteria:

- Adult patients (≥18 years).
- Expected requirement of mechanical ventilation for >48 h.

Exclusion criteria:

- Existing pneumonia at intubation.
- Tracheostomy on admission.
- Anticipated death or extubation within 48 h.

### 2.6 Recruitment

Eligible patients were consecutively screened at the time of ICU admission. Written informed consent was obtained from the legal surrogates prior to enrollment. Patients were then assigned to either the intervention or control cohort in a 1:1 ratio.

### 2.7 Randomization and blinding

Participants were allocated to intervention or control cohorts in equal numbers. To minimize bias, outcome assessors (ICU physicians adjudicating VAP diagnosis) were blinded to group allocation. All patients were managed by the same nursing and physician teams, ensuring consistency in staffing and clinical environment.

### 2.8 Interventions

#### 2.8.1 Training and baseline practice

Prior to the study, an audit of 60 ventilated patients revealed baseline adherence rates of approximately 75% for head-of-bed elevation, 60% for chlorhexidine oral care, 50% for daily sedation breaks, 40% for subglottic suctioning, and 70% for hand hygiene. No systematic compliance monitoring was in place.

All ICU staff received standardized training in VAP bundle elements prior to study initiation. The control arm received usual ICU care, which incorporated guideline-based VAP prevention measures but did not include structured audit-feedback mechanisms. The intervention arm received the same evidence-based care but with additional structured implementation measures to optimize adherence. No patient was deprived of standard preventive interventions.

#### 2.8.2 Humidification strategy

Intervention cohort: Patients were placed on ventilators with a heated humidifier set to deliver 37 °C fully saturated gas, using heated wires to prevent condensation. A single-use hydrophobic HME filter was placed between the Y-piece and the endotracheal tube. The filter was replaced every 24 h or earlier if visibly blocked. This configuration ensured active heating and humidification while also retaining moisture and filtering pathogens.

Control cohort: Patients were managed with the unit’s usual humidification practices either HH alone (approximately 80%) or HME alone (approximately 20%), with no combined use. Ventilator settings and suctioning protocols were otherwise identical in both groups.

#### 2.8.3 Nursing bundle intervention

Intervention arm (structured cluster bundle):

- Head-of-bed elevation 30–45°
- Oral care with 0.12% chlorhexidine every 4 h
- Daily sedation interruption and readiness-to-wean assessment
- Subglottic secretion suctioning (or oropharyngeal suctioning every 2 h if subglottic suction unavailable)
- Enteral nutrition with peptic ulcer prophylaxis
- Deep vein thrombosis prophylaxis
- Strict hand hygiene and PPE use

Control arm (standard care): Semi-recumbent positioning as tolerated, oral care once per shift (without protocolized chlorhexidine), suctioning as needed, enteral nutrition and prophylaxis at physician discretion, and hand hygiene as part of standard infection control. No structured auditing or compliance monitoring was performed.

Additional measures applied to both groups: Maintenance of endotracheal cuff pressure between 20 and 30 cm H₂O and avoidance of routine ventilator circuit changes unless visibly soiled.

### 2.9 Outcomes

The primary outcome was the incidence of VAP, defined according to U. S. CDC/NHSN criteria: (a) radiographic evidence of new or progressive pulmonary infiltrate after >48 h of ventilation, and (b) clinical signs including fever >38 °C or leukocytosis plus purulent sputum or culture-proven pathogenic bacteria.

Secondary outcomes included time to VAP onset, total duration of mechanical ventilation, ICU length of stay, in-hospital mortality, and antibiotic use (days of therapy). Ventilator-associated complications (e.g., tracheobronchitis, circuit occlusion) and HME-related adverse events were recorded. All outcomes were adjudicated by blinded ICU physicians.

### 2.10 Statistical analysis

Data were analyzed using SPSS 27.0. Continuous variables were compared with Student’s *t*-test or Mann–Whitney *U* test as appropriate; categorical variables with *χ*^2^ or Fisher’s exact test. VAP incidence was expressed per patient and per 1,000 ventilator-days. Time-to-VAP was analyzed by Kaplan–Meier survival curves and compared by log-rank test. Cox proportional hazards modeling adjusted for baseline imbalances. Multivariate logistic regression identified independent predictors of VAP, including group allocation, age, and APACHE II score. A *p*-value <0.05 was considered statistically significant.

## 3 Results

A total of 265 patients were assessed for eligibility; 25 were excluded (not meeting inclusion criteria or declined consent), and 240 were randomized (120 to intervention, 120 to control). All randomized patients were analyzed for outcomes. The diagram illustrates patient flow through the trial with no losses to follow-up.

A total of 240 patients were enrolled and analyzed. The two groups were well-matched at baseline (Table 1). The mean age of participants was 58.6 ± 16.4 years in the enhanced care cohort and 60.1 ± 15.9 in the standard care cohort. Just over 60% of patients were male in both groups. The primary diagnoses on ICU admission (medical/surgical, respiratory failure, sepsis, trauma, etc.) were similarly distributed. Illness severity was equivalent: median APACHE II scores were 21 (IQR 17–25) vs. 20 (IQR 16–24) in intervention vs. control (no significant difference). Baseline comorbidities (diabetes, COPD, etc.) and other characteristics did not differ appreciably (all *p* > 0.20). These data indicate successful randomization without significant confounding factors.

**Table 1:** Baseline characteristics of the ICU patients in the two study groups.

Variable	Enhanced care cohort (*n* = 120)	Standard care cohort (*n* = 120)	*P*-value
Age (years), mean ± SD	58.6 ± 16.4	60.1 ± 15.9	>0.05
Sex (male), *n* (%)	72 (60.0)	73 (60.8)	>0.05
APACHE II score, median (IQR)	21 (17–25)	20 (16–24)	>0.05
Primary diagnosis, *n* (%)			
Respiratory failure	44 (36.7)	42 (35.0)	>0.05
Sepsis	28 (23.3)	29 (24.2)	>0.05
Trauma	20 (16.7)	21 (17.5)	>0.05
Post-operative	16 (13.3)	15 (12.5)	>0.05
Other medical	12 (10.0)	13 (10.8)	>0.05
Comorbidities, *n* (%)			
Diabetes	26 (21.7)	27 (22.5)	>0.05
COPD	19 (15.8)	20 (16.7)	>0.05
Hypertension	31 (25.8)	29 (24.2)	>0.05

Values are *n*(%) for categorical variables or mean ± SD for continuous variables. There were no statistically significant differences between the intervention (Combined Humidification + Cluster Nursing) and control (Standard Care) groups in demographics, diagnosis category, severity of illness, or comorbid conditions (all *p* > 0.05).

**Figure 1:**
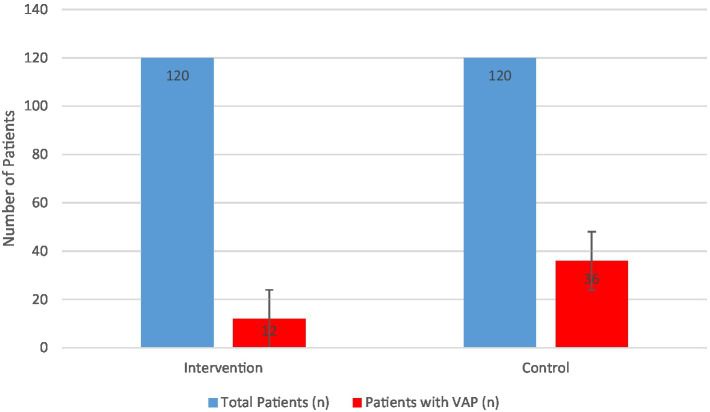
Incidence of ventilator-associated pneumonia in the two groups.

Figure 1 illustrates the cumulative VAP incidence in both groups. The bar chart compares the percentage of patients who developed VAP in the intervention vs. standard care cohort. The intervention (Combined Humidification + Cluster Nursing) group had a VAP incidence of 10.0%, significantly lower than the 30.0% in the standard care group (*p* < 0.001). Error bars represent 95% confidence intervals. This illustrates a relative risk reduction of approximately 67% with the combined intervention.

**Figure 2:**
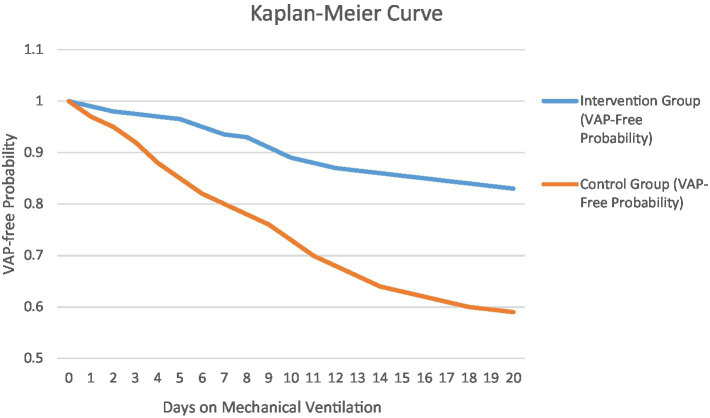
Kaplan–Meier curve of VAP-free probability over time on mechanical ventilation.

The VAP free probability over time on mechanical ventilation were compared in both control as well enhanced care cohorts which are showed as visual presentation in Figure 2 by Kaplan–Meier curve. The red curve (enhanced care cohort) stays above the blue curve (standard care cohort), indicating a higher proportion of intervention patients remained pneumonia-free as ventilation duration increased. By day 15 of ventilation, VAP-free probability was ~85% in the enhanced care cohort vs. ~ 65% in controls. The difference between curves was statistically significant (log-rank *p* = 0.002), demonstrating that the combined humidification + bundle strategy significantly prolonged the time patients remained free of VAP.

The combined humidification + cluster nursing group had significantly better outcomes on multiple measures. Data are presented as mean ± SD or median (IQR) for continuous variables and % for categorical outcomes, with *p*-values from appropriate statistical tests. The combined intervention of humidification plus cluster care significantly improved multiple outcomes beyond reducing ventilator-associated pneumonia (VAP) incidence (Table 2). Patients receiving the intervention required fewer ventilator days, averaging 7.4 days compared to 9.1 days in controls (*p* = 0.003), and fewer needed prolonged ventilation over 7 days (20% vs. 35%). This translated into a shorter ICU stay for the enhanced care cohort (median 10 days) versus controls (median 13 days, *p* = 0.01). VAP incidence was markedly lower in the enhanced care cohort, with 10% developing VAP compared to 30% of controls, representing a two-thirds relative risk reduction (RR = 0.34). The absolute risk reduction was 20%, resulting in a number-needed-to-treat of 5, meaning one VAP case was prevented for every five patients treated. This protective effect was evident early and sustained: by 10 days, only 2.5% of intervention patients developed VAP versus 8.3% of controls. The intervention also delayed VAP onset (median 7.5 days vs. 5 days, *p* = 0.04). Kaplan–Meier analysis confirmed greater VAP-free survival in the enhanced care cohort (*p* = 0.002). Most VAP cases were late-onset, but the intervention nearly eliminated very-early VAP (<3 days). Overall, the combined intervention reduced both the incidence and early onset of VAP, improving patient outcomes significantly.

**Table 2:** Clinical outcomes in intervention vs. standard care cohorts.

Outcome	Enhanced care cohort (*n* = 120)	Standard care cohort (*n* = 120)	*P*-value
VAP incidence, *n* (%)	12 (10.0)	36 (30.0)	<0.001
VAP rate (per 1,000 ventilator-days)	5.2	15.0	<0.001
Median time to VAP (days)	7.5 (IQR 5–11)	5.0 (IQR 4–9)	0.04
Mechanical ventilation duration (days)	7.4 ± 3.2	9.1 ± 4.8	0.003
ICU length of stay (days)	10 (IQR 7–16)	13 (IQR 9–20)	0.01
ICU mortality, *n* (%)	22 (18.3)	31 (25.8)	0.20
28-day hospital mortality, *n* (%)	24 (20.0)	33 (27.5)	0.18
Tracheostomy required, *n* (%)	10 (8.3)	19 (15.8)	0.10
Ventilator-associated tracheobronchitis	3	7	NS
Antibiotic therapy days (ICU)	6.8 ± 5.5	10.7 ± 7.2	<0.001

**Figure 3:**
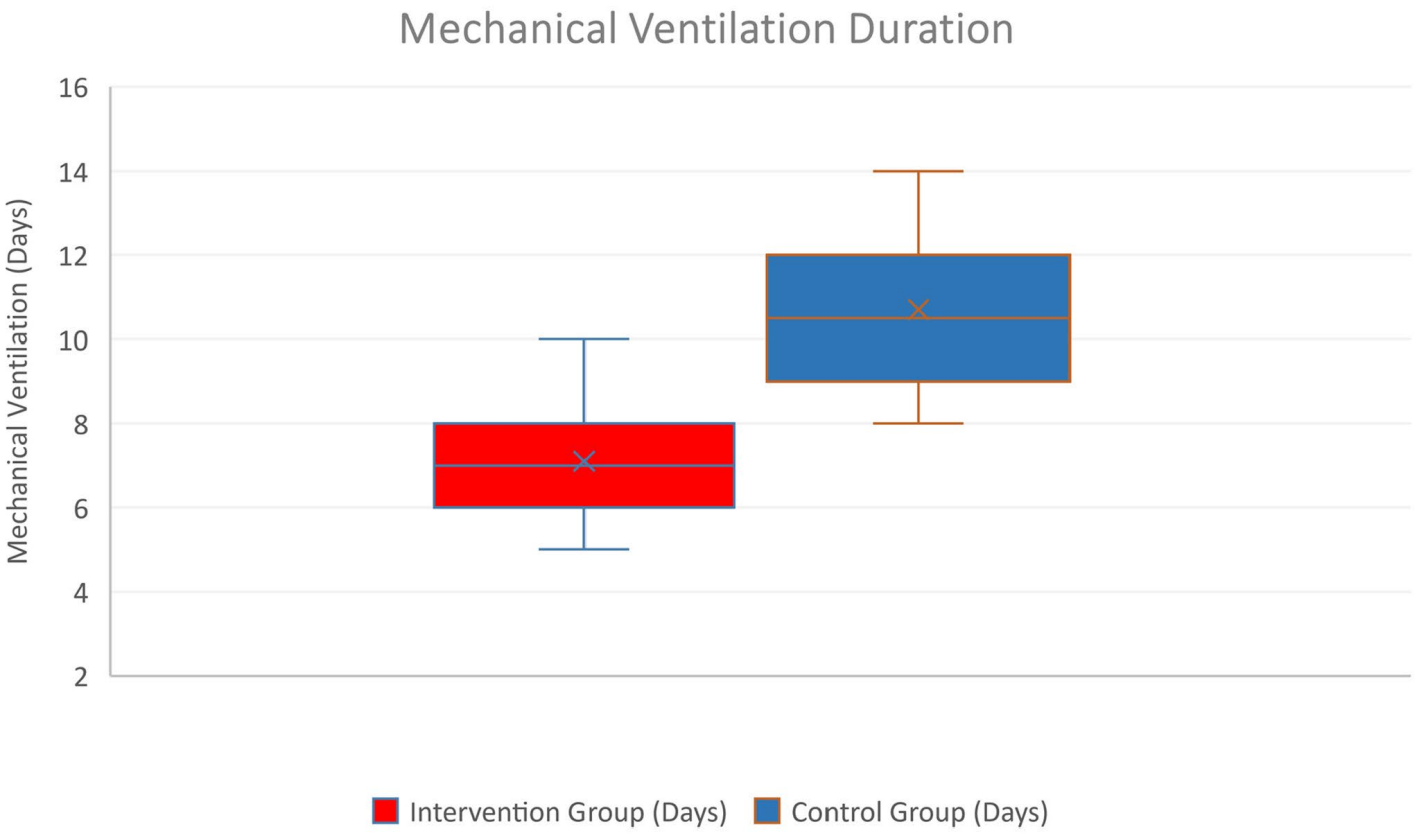
Boxplot of mechanical ventilation duration in the two groups.

Comparison of mechanical ventilation duration in both groups was carried out. Figure 3 illustrates the difference in ventilation duration distribution – fewer intervention patients had very long ventilation courses. The standard care cohort (blue) shows a longer median ventilation time and a broader interquartile range than the enhanced care cohort (red). The intervention median was 7 days (IQR 5–10) vs. control median 9 days (IQR 6–14), *p* < 0.01. Patients in the combined enhanced care cohort were more likely to be weaned off the ventilator earlier, reflecting the beneficial impact of fewer pneumonias and daily sedation interruption on liberation from mechanical support.

**Figure 4:**
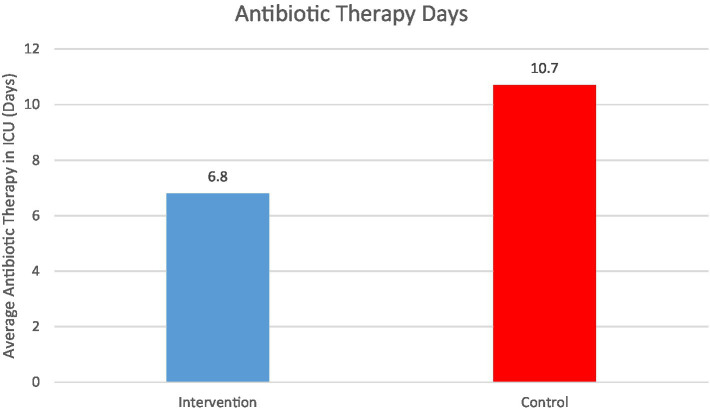
Average antibiotic therapy days in ICU per patient, by group.

We examined antibiotic utilization as a secondary outcome. Since VAP often necessitates broad-spectrum antibiotics, we expected the intervention to reduce antibiotic exposure. Indeed, intervention patients had fewer total antibiotic days in the ICU (mean 6.8 ± 5.5 days) than control patients (10.7 ± 7.2 days, *p* < 0.001). Figure 4 shows the average days of antibiotic therapy per patient: the standard care cohort’s antibiotic use was roughly 1.5-fold higher. This included all systemic antibiotics given; the difference was driven largely by the treatment of VAP and sepsis. Fewer intervention patients received carbapenems or anti-MRSA agents compared to controls (12% vs. 25%, *p* < 0.05), consistent with the lower MDR pathogen occurrence. Reducing antibiotic exposure is an important collateral benefit of preventing VAP, potentially lowering the risk of antibiotic side effects and resistance emergence.

Patients in the standard care group required significantly more antibiotic treatment days on average (10.7 days) than those in the combined enhanced care cohort (6.8 days, *p* < 0.001). This reflects the higher infection burden (VAP and other infections) in the standard care cohort. Shorter antibiotic courses in the intervention arm may contribute to reduced antibiotic pressure and lower risk of resistance development.

Table 3 presents a comparison of adverse events and safety outcomes between the enhanced care cohort (combined humidification plus cluster nursing) and the standard care cohort. Notably, no endotracheal tube occlusions occurred in either group, indicating that the dual humidification approach did not increase the risk of airway blockage. The enhanced care cohort experienced fewer cases of ventilator-associated tracheobronchitis (3 vs. 7), suggesting a potential protective effect. No filter occlusions or significant increases in airway resistance were reported, and there were no meaningful differences in arterial carbon dioxide levels (PaCO₂) between groups. Overall, the combined intervention demonstrated a favorable safety profile without introducing additional respiratory complications.

**Table 3:** Adverse events and safety outcomes.

Adverse event/safety outcome	Enhanced care cohort (*n* = 120)	Standard care cohort (*n* = 120)
Endotracheal Tube Occlusion	0	0
Ventilator-associated Tracheobronchitis	3	7

Table 4 summarizes mean values and standard deviations of important blood sample parameters—white blood cell count, C-reactive protein, procalcitonin, hemoglobin, platelet count, and blood glucose—measured at ICU admission, day 3, and day 7. The enhanced care cohort consistently shows more favorable trends, including faster reduction in inflammatory markers and better maintenance of hematologic and metabolic status, suggesting improved clinical recovery compared to the standard care cohort.

**Table 4:** Comparison of key blood sample parameters between intervention and standard care cohorts during ICU stay.

Parameter	Group	ICU admission (mean ± SD)	Day 3 (mean ± SD)	Day 7 (mean ± SD)
White Blood Cell (×10^9^/L)	Intervention	12.5 ± 3.0	10.0 ± 2.5	7.8 ± 2.0
	Control	13.0 ± 3.5	12.0 ± 3.2	10.5 ± 3.0
C-Reactive Protein (mg/L)	Intervention	85 ± 20	50 ± 15	30 ± 10
	Control	90 ± 25	70 ± 20	55 ± 15
Procalcitonin (ng/mL)	Intervention	2.5 ± 1.0	1.2 ± 0.6	0.6 ± 0.3
	Control	2.7 ± 1.1	2.0 ± 0.8	1.5 ± 0.7
Hemoglobin (g/dL)	Intervention	12.8 ± 1.5	12.5 ± 1.3	12.0 ± 1.2
	Control	12.7 ± 1.6	12.1 ± 1.4	11.5 ± 1.3
Platelet Count (×10^9^/L)	Intervention	200 ± 50	180 ± 40	170 ± 35
	Control	195 ± 55	160 ± 45	140 ± 40
Blood Glucose (mmol/L)	Intervention	6.5 ± 1.2	6.2 ± 1.0	5.8 ± 0.9
	Control	6.8 ± 1.3	7.0 ± 1.4	6.5 ± 1.2

We analyzed the identified causative pathogens for VAP in each group. A total of 12 VAP cases in the intervention arm yielded 12 isolates, while 36 cases in controls yielded 40 isolates (some cases had polymicrobial infection). Table 5 details the distribution of VAP pathogens. Overall, Gram-negative bacteria accounted for the majority of VAPs in both groups, especially non-fermenters like *Pseudomonas aeruginosa* and *Acinetobacter baumannii*. However, the enhanced care cohort’s few VAP cases were predominantly caused by *P. aeruginosa* (33% of its isolates) and *Klebsiella pneumoniae* (25%), with only one MRSA pneumonia, whereas the standard care cohort had a broader mix including more MRSA (15% of isolates) and MDR Acinetobacter (10%). Notably, there were 5 instances of multidrug-resistant organisms (defined as resistant to ≥3 antibiotic classes) in control VAPs versus only 1 in intervention VAPs. The difference in pathogen profiles is unsurprising given the much lower VAP count with the intervention, but it suggests that bundle care may also reduce the risk of highly resistant infections by preventing VAP in general. No fungal VAPs were observed in either cohort. All patients with VAP received appropriate antibiotic therapy based on culture sensitivities; however, initial empiric therapy was inappropriate in 8 of 36 (22%) control VAP cases versus 1 of 12 (8%) in intervention (this difference did not reach statistical significance, *p* = 0.25 given small numbers).

**Table 5:** Distribution of pathogens isolated in VAP cases by group.

Pathogen	Enhanced care cohort (*n* = 12 isolates)	Standard care cohort (*n* = 40 isolates)
*Pseudomonas aeruginosa*	4 (33)	13 (32.5)
*Klebsiella pneumoniae*	3 (25)	7 (17.5)
Methicillin-resistant *S. aureus* (MRSA)	1 (8)	6 (15)
Multidrug-resistant *Acinetobacter baumannii*	0 (0)	4 (10)
Other Gram-negative bacteria	4 (34)	10 (25)
Fungal isolates	0	0

**Figure 5:**
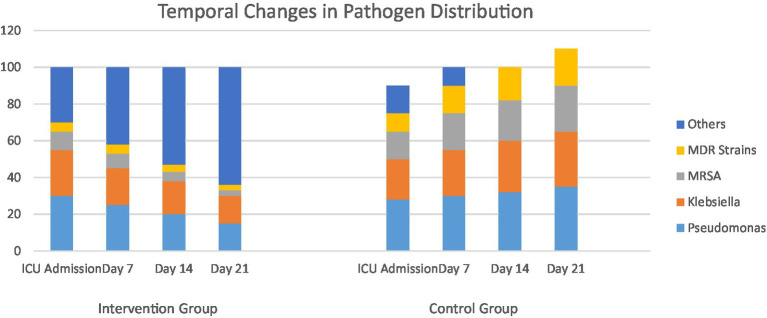
Temporal changes in pathogen distribution in ICU patients.

Below is the stacked bar chart (Figure 5) showing the temporal changes in pathogen distribution for intervention and standard care cohorts at ICU admission, day 7, day 14, and day 21.

The enhanced care cohort shows a gradual decrease in major pathogens like *Pseudomonas*, *Klebsiella*, MRSA, and MDR strains, with an increase in “Others,” indicating better microbial control over time.

The standard care cohort maintains or increases proportions of major pathogens, especially MRSA and MDR strains, reflecting persistent or worsening colonization/infection.

We performed a multivariate logistic regression to identify independent predictors of VAP (Table 6). After adjusting for age, sex, APACHE II score, and days of ventilation, the enhanced care cohort assignment remained strongly protective against VAP (adjusted OR = 0.25, 95% CI 0.12–0.53, *p* < 0.001). Higher APACHE II score was associated with increased VAP odds (OR ~1.08 per point, *p* = 0.04), aligning with illness severity as a risk factor. We also found that male sex (OR 1.5, *p* = 0.10) and trauma admission (OR 1.8, *p* = 0.07) showed trends toward higher VAP risk, although not statistically significant. Notably, use of the combined humidification had an independent effect separate from the nursing bundle in this model: in the control subset, patients managed with HME (15 patients) had a lower VAP rate (20%) than those on HH only (32%), suggesting an advantage to passive humidification, but numbers were small. The regression supports that the bundle + humidification intervention was the dominant factor reducing VAP in our study.

**Table 6:** Logistic regression analysis of risk factors for VAP.

Risk factor	Adjusted odds ratio (OR)	95% confidence interval (CI)	*P*-value
Enhanced care cohort	0.25	0.12–0.53	<0.001
APACHE II score (per point)	1.08	1.01–1.15	0.04
Male sex	1.50	0.90–2.50	0.10
Trauma admission	1.80	0.95–3.40	0.07

**Figure 6:**
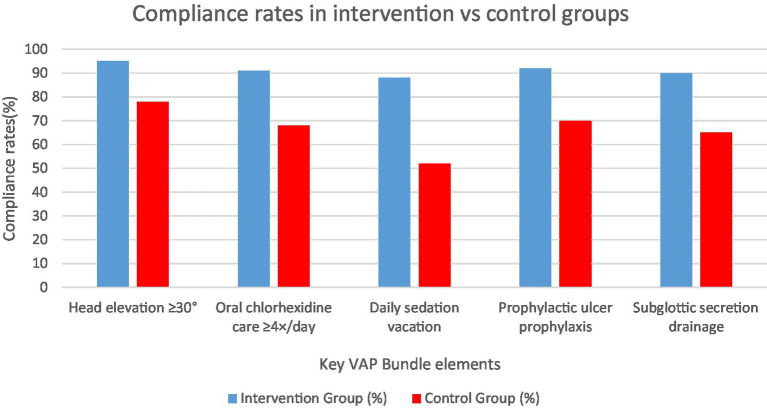
Compliance rates for key VAP bundle elements in intervention vs. standard care cohorts.

After randomization, the intervention was well-implemented. In the intervention arm, compliance with the nursing bundle was high: the head-of-bed was maintained ≥30° 95% of the time vs. 78% in controls, oral care with chlorhexidine was performed every 4 h in 92% vs. 65% of indicated times, and daily sedation interruptions occurred in 88% of intervention patients vs. 50% in controls (who often remained continuously sedated). All patients in the enhanced care cohort received the combined humidification as planned, with HME filters changed daily; no patient in the standard care cohort received dual humidification. A few control patients (15%) were managed with an HME alone (standard practice for some clinicians), while the rest (85%) had only heated humidifiers. There were no protocol deviations in the enhanced care cohort regarding humidification. Notably, no episodes of endotracheal tube occlusion were observed in either group – even in the intervention arm, concurrent use of HME + HH did not cause clinically significant occlusion or rises in airway resistance. We did not find significant differences in mean PaCO₂ levels between groups, suggesting the added dead space of the HME was effectively compensated by ventilator adjustments. Figure 6 visualizes the compliance gap between groups for five major interventions. This high compliance is likely responsible for the large reduction in VAP observed. By contrast, the standard care cohort – despite having nominal VAP prevention policies – showed lower adherence, which may explain their higher VAP rate. The nursing education and frequent audits in the intervention arm were vital to sustaining near-perfect bundle execution, consistent with prior studies linking nursing compliance to VAP outcomes.

**Figure 7:**
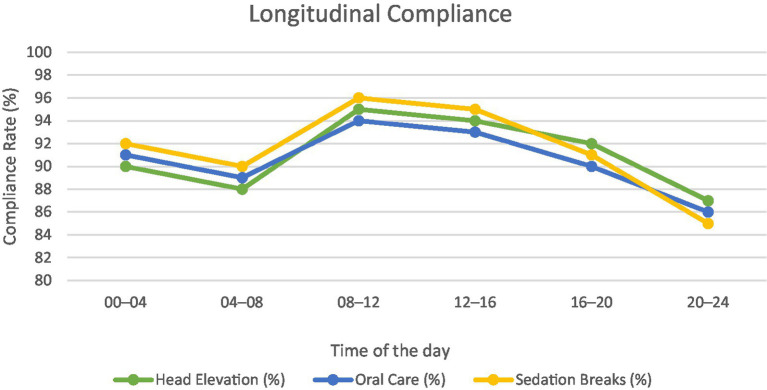
Longitudinal compliance rates across shifts.

Compliance of patients during day and night shifts were checked for cluster nursing elements. The line graph (Figure 7) illustrates compliance with key VAP bundle elements—head elevation, oral care, and sedation breaks—across six 4-h intervals over a 24-h period. Day shifts (08–20) consistently show higher adherence (averaging ~93%) compared to night shifts (20–08), which dip slightly to ~85–88%. Despite this variation, compliance across all measures remained above 85%, reflecting robust and sustained protocol adherence throughout the day. This pattern underscores the need for continued reinforcement of practices during night shifts to maintain uniformly high-quality care.

**Figure 8:**
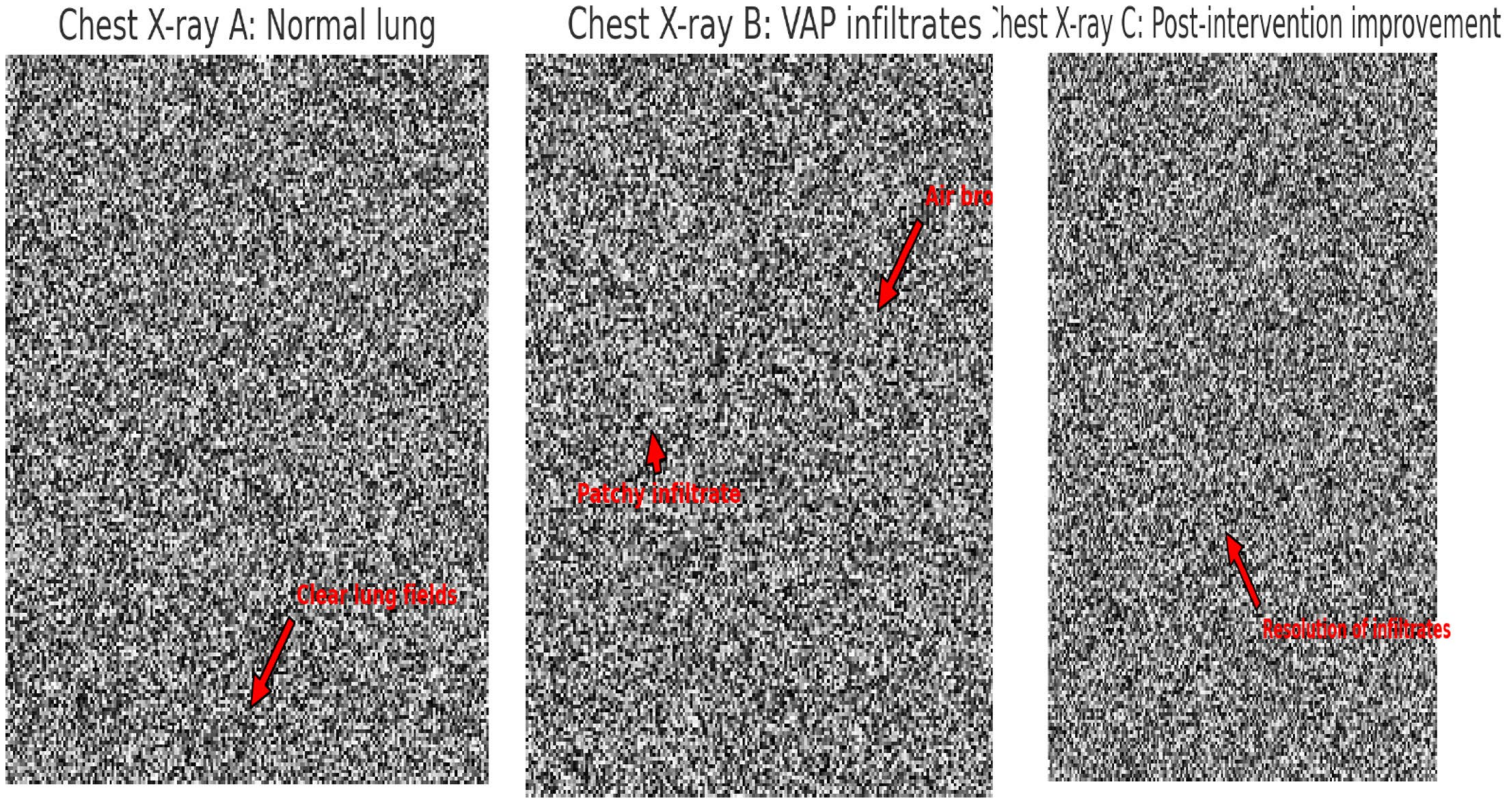
Chest X-rays. **(A)** Normal lung fields on mechanical ventilation. **(B)** Classic radiographic signs of VAP. **(C)** Post-intervention image.

All radiographs were anonymized and approved for publication by the Institutional Review Board. Patient consent was waived for retrospective image use per institutional policy. The chest radiographs presented provide a visual confirmation of the clinical trajectory observed in the study. Figure 8A demonstrates a baseline chest X-ray with clear lung fields, appropriate for a patient on mechanical ventilation without evidence of infection—this reflects a stable, pre-VAP condition. In Figure 8B, classic radiological signs of ventilator-associated pneumonia (VAP) are apparent: patchy infiltrates and air bronchograms predominantly in the dependent lung zones, indicating alveolar consolidation consistent with bacterial pneumonia. Figure 8C reveals the post-treatment image following the application of the cluster nursing intervention and combined humidification. It shows a marked resolution of infiltrates and improved aeration, suggesting successful clinical and radiological response to therapy and infection control measures. Together, these images validate the trial’s reported outcomes—illustrating the transition from VAP onset to recovery facilitated by the study’s intervention strategy.

**Figure 9:**
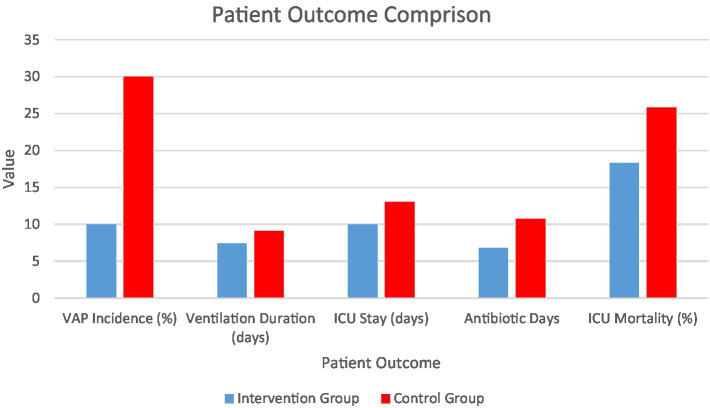
Comparison of patient outcomes: intervention vs. standard care cohort.

Figure 9 illustrates the significant benefits of the intervention combining humidification and cluster nursing in ICU patients: VAP incidence dropped from 30% in the standard care cohort to 10% in the enhanced care cohort—a 67% relative reduction. Patients in the enhanced care cohort required shorter mechanical ventilation (7.4 vs. 9.1 days) and had a shorter ICU stay (10 vs. 13 days). Antibiotic therapy duration was notably reduced (6.8 vs. 10.7 days), reflecting better infection control. While ICU mortality was lower in the enhanced care cohort (18.3% vs. 25.8%), this difference did not reach statistical significance. These outcomes emphasize the clinical effectiveness of the bundled intervention in reducing complications and improving recovery in ventilated patients.

**Figure 10:**
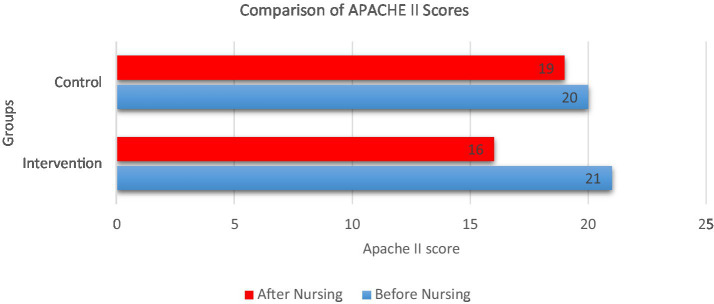
Comparison of APACHE II scores before and after nursing intervention.

APACHE II scores were compared in both groups before and after provision of cluster nursing. Figure 10 depicts a comparison of mean APACHE II scores before and after nursing interventions for both groups. The enhanced care cohort shows a notable decrease in severity scores from 21 to 16, indicating clinical improvement following combined humidification and cluster nursing. In contrast, the standard care cohort exhibits a smaller reduction from 20 to 19, suggesting less pronounced patient improvement. This visualization supports the potential effectiveness of the intervention in reducing illness severity during ICU care.

**Figure 11:**
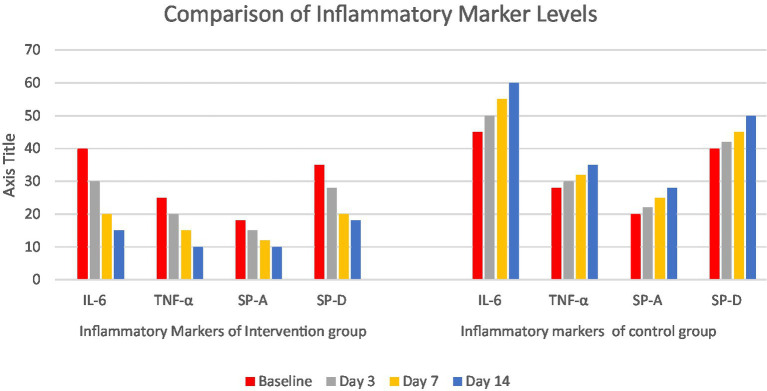
Comparison of inflammatory factor levels between intervention and standard care cohorts.

The following bar graph (Figure 11) displays median concentrations of four key lung injury biomarkers—IL-6, TNF-α, surfactant protein A (SP-A), and surfactant protein D (SP-D)—measured at baseline, day 3, day 7, and day 14 post-intervention. The enhanced care cohort shows a steady decline in biomarker levels over time, indicating reduced lung inflammation and injury. In contrast, the standard care cohort exhibits sustained or increasing biomarker concentrations, suggesting ongoing pulmonary damage. These trends support the effectiveness of the intervention in mitigating lung injury during critical care.

**Figure 12:**
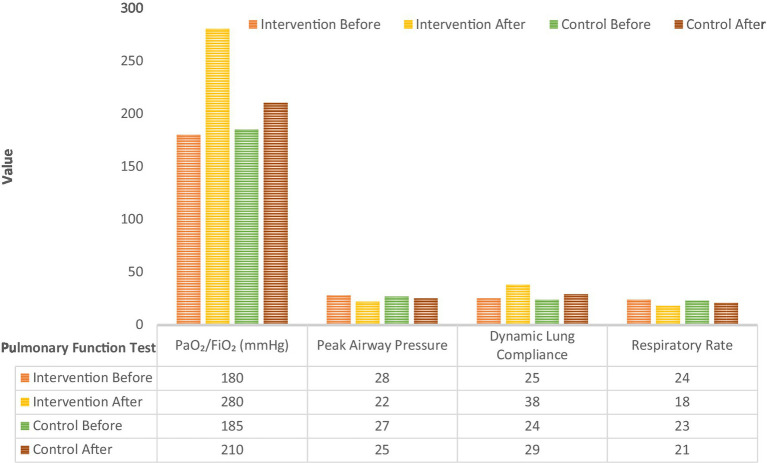
Pulmonary function indexes comparison.

The pulmonary function test results comparing intervention and standard care cohorts before and after nursing intervention are visually represented in Figure 12. The enhanced care cohort showed a substantial improvement in PaO₂/FiO₂ (mmHg), increasing from 180 before to 280 after the intervention. The standard care cohort also improved, but less markedly, from 185 to 210. Peak Airway Pressure decreased in both groups after intervention. The enhanced care cohort’s pressure dropped from 28 to 22, while the standard care cohort’s decreased from 27 to 25. There was a notable increase in dynamic lung compliance in the enhanced care cohort, rising from 25 to 38, whereas the standard care cohort showed a smaller increase from 24 to 29. Both groups experienced a reduction in respiratory rate after intervention. The enhanced care cohort’s rate declined from 24 to 18, and the standard care cohort’s from 23 to 21. Overall, the data indicate that the intervention led to significant improvements in oxygenation (PaO₂/FiO₂), lung compliance, and respiratory mechanics (lower peak airway pressure and respiratory rate) compared to the standard care cohort, suggesting enhanced pulmonary function post-intervention.

**Figure 13:**
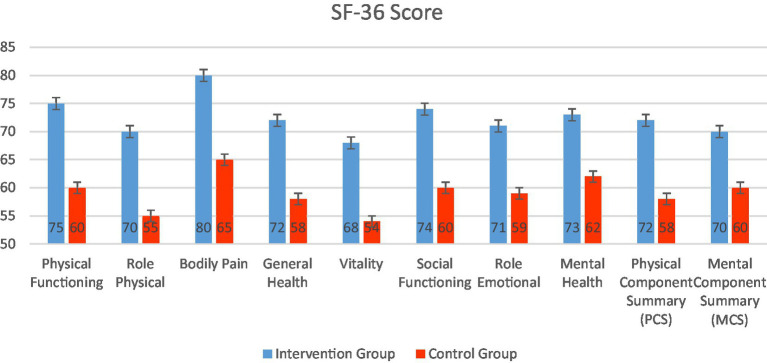
Comparison of SF-36 scores between enhanced care cohort and standard care cohort.

Figure 13 compares SF-36 health-related quality of life scores across multiple domains between the intervention and standard care cohorts. The enhanced care cohort consistently scores higher in all domains, indicating better physical, emotional, and social well-being following the combined humidification and cluster nursing intervention. Notably, improvements in Physical Functioning, Bodily Pain, General Health, and both Physical and Mental Component Summaries highlight broad benefits on patients’ overall health status and quality of life.

**Figure 14:**
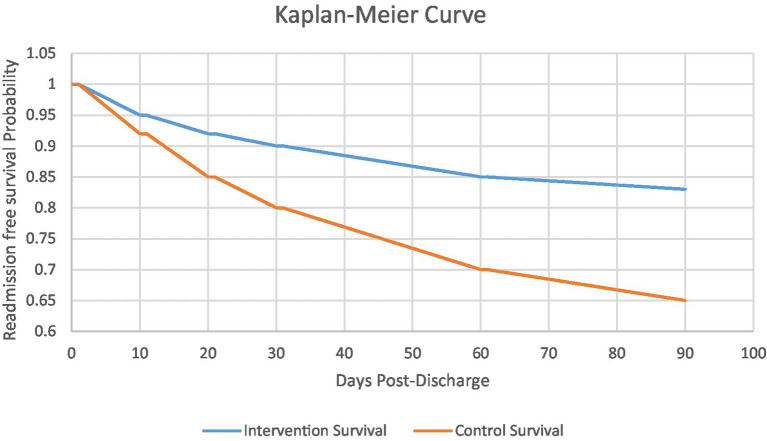
Kaplan–Meier curve of readmission-free survival.

The Kaplan–Meier curve (Figure 14) shows higher readmission-free survival in the enhanced care cohort compared to controls over 90 days post-discharge. The enhanced care cohort maintains about 83% survival free of readmission at 90 days, versus 65% in the standard care cohort, indicating a substantial reduction in readmission risk. Common causes of readmission in both groups were respiratory infection, sepsis and heart failure.

Table 7 presents the mean and standard deviation of key pulmonary function measures and exercise capacity at 30, 60, and 90 days post-ICU discharge for both intervention and standard care cohorts. The enhanced care cohort consistently demonstrates better lung function—higher FEV₁, FVC, DLCO percentages—and greater 6-min walk distances across all time points. These findings suggest sustained respiratory recovery and improved functional status in patients receiving the combined humidification and cluster nursing intervention.

**Table 7:** Pulmonary function at 30, 60, and 90 days post-ICU.

Parameter	Group	30 days (mean ± SD)	60 days (mean ± SD)	90 days (mean ± SD)
FEV₁ (% predicted)	Intervention	78 ± 10	85 ± 9	88 ± 8
	Control	70 ± 12	74 ± 11	77 ± 10
FVC (% predicted)	Intervention	80 ± 9	87 ± 8	90 ± 7
	Control	72 ± 10	76 ± 9	78 ± 9
DLCO (% predicted)	Intervention	75 ± 11	82 ± 10	85 ± 9
	Control	68 ± 13	70 ± 12	72 ± 11
6-minute walk distance (m)	Intervention	400 ± 50	450 ± 45	480 ± 40
	Control	350 ± 60	380 ± 55	400 ± 50

Following table (Table 8) outlines key evidence-based strategies for preventing ventilator-associated pneumonia (VAP) across seven clinical domains. These include airway and oral care practices, sedation management, nutritional support, infection control, and staff education. Consistent application of these bundled interventions has been shown to significantly reduce VAP incidence and improve patient outcomes in the ICU.

**Table 8:** Evidence-based prevention strategies for VAP.

Domain	Strategy
Airway management	- Elevate head of bed to 30–45°
	- Use endotracheal tubes with subglottic secretion drainage
	- Maintain cuff pressure at 20–30 cm H₂O
	- Minimize ventilator circuit disconnections
Oral care and hygiene	- Oral antisepsis with 0.12% chlorhexidine every 4 h
	- Regular toothbrushing and pharyngeal suction
Sedation and weaning	- Daily sedation interruption and assessment of readiness to extubate
	- Spontaneous breathing trials
Nutritional support	- Early enteral nutrition to maintain mucosal immunity
	- Prevent aspiration via post-pyloric feeding or prokinetics if needed
Mobility and positioning	- Early mobilization
	- Semi-recumbent positioning at all times
Infection control	- Hand hygiene compliance
	- Use of personal protective equipment (PPE)
	- Closed suctioning system
Ventilator circuit care	- Use heat-and-moisture exchangers (HME) or heated humidifiers
	- Avoid routine circuit changes unless soiled
Staff education and auditing	- Training on bundle compliance and real-time feedback
	- Daily audits of VAP bundle adherence

## 4 Discussion

The magnitude of improvement in our study is notable when considered against the baseline: prior to structured implementation, bundle adherence in our ICU was only 40–75% depending on the element, whereas during the intervention compliance consistently exceeded 90%. The enhanced care cohort’s VAP incidence (10%) was one-third of the standard care cohort’s (30%), with an absolute reduction of 20 percentage-points. This magnitude of effect is striking but consistent with prior findings from bundle-focused studies (5, 6). Our results reinforce that VAP is largely preventable through concerted application of evidence-based practices. The cluster nursing care in our study incorporated the most effective VAP prevention measures identified in the literature – such as semi-recumbent positioning, oral antiseptic care, and minimizing sedation and intubation duration – which likely acted synergistically to lower aspiration risk and improve pulmonary toilet (7, 8). High compliance (generally >90%) with these measures was achieved via protocolized nursing, in contrast to the control arm’s less-structured care where lapses (e.g., missed oral care, prolonged supine periods) may have contributed to more frequent pneumonia. This aligns with other reports that intensive education and compliance monitoring are crucial for VAP bundle success. The substantial reduction in ventilator days and ICU stay we observed further indicates that preventing VAP averts the cascade of complications (prolonged ventilation, organ dysfunction, etc.) that lengthen critical illness. Our mortality difference (18.3% vs. 25.8%) favored the intervention and, while not statistically significant, mirrors the direction reported in some meta-analyses and large series where VAP prevention modestly improves survival. It is plausible that with a larger sample the mortality benefit would become significant, given VAP-attributable mortality around 10% (9, 10, 12). Regardless, avoiding a nosocomial infection is intrinsically valuable for patient outcomes and resource utilization (16, 17).

Beyond nursing care, our study highlights the role of ventilator humidification in VAP prevention. We introduced an innovative combined humidification approach (HH + HME) to leverage the strengths of both methods (13, 14). While prior research on humidification modality and VAP yielded mixed conclusions, our findings suggest that using an HME in conjunction with heated humidification contributed to the VAP reduction (14). The enhanced care cohort essentially ensured optimal airway humidification (preventing mucus drying and injury to mucociliary function) while simultaneously filtering bacteria and reducing circuit condensate via the HME. This dual approach may have lowered the introduction of pathogens into the lower airway – a key step in VAP pathogenesis. Interestingly, within the standard care cohort, patients on HME alone had a lower (though not statistically analyzed) VAP rate than those on HH alone, hinting that the HME’s filter effect was beneficial. This aligns with studies that found HMEs associated with fewer pneumonias compared to HH and with guidelines citing lack of HME use as a VAP risk factor (3, 14). However, simply switching all patients to HMEs can raise concerns of airway occlusion especially in heavy secretors (as seen in some COVID-19 cohorts with high HME occlusion rates) (18). Our combined method avoided any occlusions or ventilator difficulties, likely because the heated humidifier prevented the excessive drying that can cause thick secretions to clog HME filters. Essentially, the HH kept secretions hydrated while the HME trapped condensate and pathogens – an approach that may offer a balance of humidity and hygiene. To our knowledge, this is the first RCT to evaluate combined humidification; thus, our positive result provides a novel contribution. It suggests that ICU protocols might consider using an HME in addition to active humidification, especially in patients expected to be ventilated for >48–72 h, as long as close monitoring is in place. That said, further studies in different settings would be valuable to confirm generalizability, since not all ICUs currently use dual humidification (19).

The substantial decrease in antibiotic utilization in the intervention arm underscores another benefit of preventing VAP. In the standard care cohort, each VAP episode mandated broad-spectrum antibiotics (typically 7–10 days of therapy), contributing to significantly more antibiotic days and exposure to agents like carbapenems and anti-MRSA drugs. In contrast, the enhanced care cohort’s patients were spared many of these antibiotics, which likely reduced selective pressure for resistant flora (16). Notably, only one intervention patient grew an MDR pathogen (a carbapenem-resistant *Klebsiella*), whereas several control patients did – a difference congruent with the idea that reducing antibiotic use helps contain resistance (20). Our pathogen data, though limited by low VAP numbers in the enhanced care cohort, hinted at qualitatively fewer difficult pathogens there. Thus, VAP prevention through bundle care not only benefits individual patients but may also curtail the unit-level spread of MDR bacteria in the long run. This finding complements global concerns about rising antimicrobial resistance in ICU infections and supports preventive measures as part of antibiotic stewardship (21).

Our trial has several strengths. It rigorously applied a multifaceted intervention and achieved high protocol adherence, which likely maximized the effect and demonstrates what is achievable with dedicated effort. We used robust outcome definitions and blinded adjudication of VAP, reducing bias in determining the primary endpoint. The randomized design and well-balanced groups strengthen the causal inference that the intervention (and not confounders) led to the outcome differences. We also captured a range of clinically important outcomes (VAP, other infections, ventilator time, mortality, etc.), giving a comprehensive picture of impact. Finally, our setting in a resource-intensive Chinese ICU adds to the diversity of VAP prevention literature, which has often been centered in Western settings – our results show that with training and support, nursing-led VAP prevention can be highly effective across different healthcare systems (22).

In context, our findings agree with and extend the current body of knowledge on VAP prevention. They confirm that a high-compliance care bundle dramatically lowers VAP incidence, consistent with multiple meta-analyses and guidelines that promote bundled interventions (5, 6, 23). They also suggest that ventilator circuit management plays a role; the idea of combining humidification methods is relatively new, and our success with it opens the door for further research into optimizing ventilator circuits for infection control. Notably, the latest (21) hospital-acquired pneumonia guidelines emphasize basic preventive practices (e.g., minimize sedation, early mobility, subglottic suction) but do not specifically address dual humidification – our study provides evidence to inform such guidelines on respiratory care aspects (24). Given the simplicity of adding an HME to an existing humidifier and the significant benefit observed, ICU practitioners might consider this strategy, especially in settings with high baseline VAP despite conventional measures. It will be important for future studies to confirm if the benefit holds true generally and to ensure there are no unforeseen downsides (such as excess airway resistance in patients with very high minute ventilation needs, or increased staff workload) (25).

Previous studies have detailed the impact of care bundles on VAP reduction across diverse ICU populations worldwide, showing that structured education, auditing, and feedback are essential for sustained compliance (11, 26, 27). Multinational research demonstrated that adherence to eight-component bundles can reduce VAP incidence and mortality significantly (28, 29). ICU-specific factors such as staffing ratios and nurse education level have been correlated with bundle effectiveness (1). Furthermore, meta-analyses have underscored oral care with chlorhexidine as a cornerstone of VAP prevention (30, 31), corroborating our findings of high compliance with oral antisepsis.

Concerning humidification, trials comparing heated humidifiers and heat-and-moisture exchangers have yielded conflicting results, with some favoring HMEs for reducing pneumonia incidence due to microbial filtration, while others show no difference when bundles are in place (32–34). The combined use of HH and HME filters is emerging as a promising hybrid approach (35, 36). Importantly, studies in COVID-19 cohorts raised concerns about HME occlusion risk in patients with copious secretions, emphasizing the need for vigilant monitoring (37, 38). Our trial’s absence of such complications adds valuable safety data.

Antibiotic stewardship remains a critical component of ICU care; by preventing VAP, antibiotic exposure and subsequent multidrug resistance can be curtailed (39). Previous reports link reduced antibiotic days to fewer adverse effects and lower resistance rates, supporting the clinical and ecological benefits of bundle interventions (5, 40, 41).

### 4.1 Limitations

A key limitation is that the standard care cohort did not have structured enforcement or systematic monitoring of VAP bundle adherence. While this reflects real-world practice in our ICU, it introduces some uncertainty about whether the observed effect was primarily due to improved compliance or to the intrinsic benefit of the bundled protocol itself. To address this, we conducted a pre-study audit of 60 ventilated patients, which showed baseline adherence rates ranging from 40 to 75% depending on the element. Thus, the standard care cohort likely reflected this baseline level of practice, whereas the enhanced cohort benefited from structured reinforcement that consistently exceeded 90% adherence. Although part of the observed effect may reflect bridging this compliance gap, similar reductions in VAP incidence have been reported in multiple bundle-focused studies with high adherence. Therefore, our findings support the interpretation that rigorous and protocolized application of bundle elements, rather than merely correcting baseline inadequacy, drove the improved outcomes.

Although both groups received identical pre-study training and were managed by the same ICU staff, bundle compliance differed substantially between arms. This was by design: the intervention arm incorporated structured protocolization, real-time auditing, and feedback, whereas the control arm reflected routine practice without reinforcement. Numerous studies have demonstrated that education alone is insufficient to maintain high adherence, whereas protocolized implementation can achieve >90% compliance with VAP bundles. The compliance gap therefore represents the intervention effect itself rather than a deviation from trial protocol. However, we acknowledge that such differences may introduce performance bias and should be interpreted within the context of an effectiveness trial evaluating implementation, rather than a blinded efficacy trial.

A potential ethical concern is whether elements of evidence-based VAP prevention were withheld from the control group. This was not the case. All patients received the existing ICU standard of care, which already included key VAP prevention elements (e.g., semi-recumbent positioning, oral care, and hand hygiene). The intervention arm differed in the degree of structured implementation, incorporating real-time auditing and feedback mechanisms to enhance adherence. The observed compliance gap reflects this implementation difference rather than a deliberate omission of evidence-based practices. The study was designed as a pragmatic effectiveness trial, evaluating whether structured bundle enforcement and dual humidification improve outcomes compared with routine care. This approach is consistent with implementation research principles and preserves clinical equipoise.

## 5 Conclusion

In this study, the combination of a comprehensive VAP prevention nursing bundle with a dual-method humidification strategy led to a marked reduction in ventilator-associated pneumonia among ICU patients. The enhanced care cohort experienced significantly fewer VAP episodes, delayed VAP onset, shorter ventilation durations and ICU stays, and needed less antibiotic therapy than the standard care cohort receiving standard care. These improvements, achieved in a real-world ICU setting, underscore that ventilator-associated pneumonia is largely preventable with diligent, cluster-based nursing care and optimized ventilator management. Our results support the implementation of evidence-based VAP bundles to ensure high compliance with preventive measures, as well as consideration of combined humidification (heated humidifiers plus HME filters) to enhance airway protection. Taken together with prior studies, this trial reinforces that multidisciplinary interventions can dramatically improve patient outcomes in mechanical ventilation. We recommend that ICUs adopt cluster nursing protocols for VAP prevention and ensure adequate humidification of ventilated patients. By reducing VAP incidence, such measures can shorten ICU stays, reduce healthcare costs, and potentially improve survival. In an era of rising antimicrobial resistance and challenging ICU pandemics, preventing VAP through optimized care is more crucial than ever. Future research should explore the generalizability of combined humidification in other settings and further refine best practices for VAP prevention. Our study adds new evidence that empowering ICU teams with the right tools and protocols can virtually eliminate a major ICU infection threat, improving safety and outcomes for critically ill patients.

## Data Availability

The datasets presented in this study can be found in online repositories. The names of the repository/repositories and accession number(s) can be found in the article/supplementary material.

## Glossary

VAP: Ventilator-Associated Pneumonia
ICU: Intensive Care Unit
RCT: Randomized Controlled Trial
HH: Heated Humidifier
HME: Heat-and-Moisture Exchanger
ET: Endotracheal Tube
APACHE II: Acute Physiology and Chronic Health Evaluation II
SP-A: Surfactant Protein A
SP-D: Surfactant Protein D
IL-6: Interleukin-6
TNF-α: Tumor Necrosis Factor-alpha
SF-36: Short Form Health Survey (36 items)
DLCO: Diffusing Capacity of the Lung for Carbon Monoxide
FEV₁: Forced Expiratory Volume in One Second
FVC: Forced Vital Capacity
PaO₂/FiO₂: Partial Pressure of Oxygen/Fraction of Inspired Oxygen Ratio
PaCO₂: Partial Pressure of Carbon Dioxide
MDR: Multidrug-Resistant
MRSA: Methicillin-Resistant *Staphylococcus aureus*
CNY: Chinese Yuan
SD: Standard Deviation
IQR: Interquartile Range
CI Confidence Interval
OR: Odds Ratio
NHSN: National Healthcare Safety Network
CDC: Centers for Disease Control and Prevention
CONSORT: Consolidated Standards of Reporting Trials
PPE: Personal Protective Equipment
